# In Silico Analysis, Anticonvulsant Activity, and Toxicity Evaluation of Schisandrin B in Zebrafish Larvae and Mice

**DOI:** 10.3390/ijms241612949

**Published:** 2023-08-18

**Authors:** Dorota Nieoczym, Nancy Saana Banono, Katarzyna Stępnik, Agnieszka A. Kaczor, Przemysław Szybkowski, Camila Vicencio Esguerra, Wirginia Kukula-Koch, Kinga Gawel

**Affiliations:** 1 Department of Animal Physiology and Pharmacology, Institute of Biological Sciences, Faculty of Biology and Biotechnology, Maria Curie-Skłodowska University, Akademicka 19, 20-033 Lublin, Poland; 2 Chemical Neuroscience Group, Centre for Molecular Medicine Norway, University of Oslo, Gaustadalleen 21, Forskningsparken, 0349 Oslo, Norway; n.s.banono@ncmm.uio.no (N.S.B.); c.v.esguerra@ncmm.uio.no (C.V.E.); 3 Department of Physical Chemistry, Institute of Chemical Sciences, Faculty of Chemistry, Maria Curie-Skłodowska University, Pl. M. Curie-Skłodowskiej 3/243, 20-031 Lublin, Poland; katarzyna.stepnik@mail.umcs.pl; 4 Department of Synthesis and Chemical Technology of Pharmaceutical Substances with Computer Modeling Laboratory, Faculty of Pharmacy, Medical University of Lublin, 4A Chodzki St., 20-093 Lublin, Poland; agnieszka.kaczor@umlub.pl; 5 Department of Experimental and Clinical Pharmacology, Medical University of Lublin, Jaczewskiego St. 8b, 20-090 Lublin, Poland; pszybki@gmail.com; 6 Clinical Provincial Hospital No. 2 St. Jadwiga Krolowej in Rzeszow, Lwowska St. 60, 35-301 Rzeszow, Poland; 7 Department of Pharmacognosy with Medicinal Plants Garden, Medical University of Lublin, Chodźki St. 1, 20-093 Lublin, Poland; virginia.kukula@gmail.com

**Keywords:** schisandrin B, seizures, toxicity, zebrafish, mice, pentylenetetrazole, local field potential recordings, molecular modeling

## Abstract

The aim of this study is to evaluate the anticonvulsant potential of schisandrin B, a main ingredient of *Schisandra chinensis* extracts. Schisandrin B showed anticonvulsant activity in the zebrafish larva pentylenetetrazole acute seizure assay but did not alter seizure thresholds in the intravenous pentylenetetrazole test in mice. Schisandrin B crosses the blood–brain barrier, which we confirmed in our in silico and in vivo analyses; however, the low level of its unbound fraction in the mouse brain tissue may explain the observed lack of anticonvulsant activity. Molecular docking revealed that the anticonvulsant activity of the compound in larval zebrafish might have been due to its binding to a benzodiazepine site within the GABA_A_ receptor and/or the inhibition of the glutamate NMDA receptor. Although schisandrin B showed a beneficial anticonvulsant effect, toxicological studies revealed that it caused serious developmental impairment in zebrafish larvae, underscoring its teratogenic properties. Further detailed studies are needed to precisely identify the properties, pharmacological effects, and safety of schisandrin B.

## 1. Introduction

Epilepsy is a common and serious neurological disease characterized by spontaneous and unpredictable seizures. The reason for these symptoms is the abnormal excessive and synchronous activity of neurons in the brain caused by an imbalance between inhibitory and excitatory neurotransmission, ion channel dysfunctions (mainly K^+^ and Na^+^ channels), and/or genetic mutations. Approximately 75 million people worldwide are affected by epilepsy [1] with about 50–60% of them also having other neuropsychiatric ailments, mainly anxiety, depression, and cognitive disorders [2,3,4,5]. The pathophysiological mechanism between epilepsy and other neuropsychiatric problems seems to be bidirectional because some patients might suffer from psychiatric disorders prior to epilepsy development. Moreover, some clinical observations showed that epileptic disorders in particular brain regions might provoke anxiety attacks [2] and, on the other hand, some stressful life events might trigger epilepsy onset; anxiety and stressful incidents are identified as some of the most frequent factors that initiate seizures [3]. The coexistence of both disorders, as well as their close link, might be explained by the involvement of the same neurotransmitter systems, mainly γ-aminobutyric acid (GABA), as well as serotoninergic, dopaminergic, and adrenergic ones [2].

Both epilepsy itself, as well as comorbid neuropsychiatric disorders, must be appropriately treated, and emphasis should not only be placed on the achievement of seizure remission, but also on the treatment of other comorbidities to improve the quality of patients’ lives as much as possible. Despite at least the partial understanding of the mechanisms of epilepsy and the availability of numerous antiseizure medications (ASMs) on the pharmaceutical market, its therapy still encounters many difficulties. Firstly, pharmacotherapy with available anticonvulsant drugs, whether as mono- or polytherapy, does not present the expected therapeutic effects in about 30% of patients and are therefore considered pharmacoresistant [6,7]. Secondly, anticonvulsant drugs provide only symptomatic treatment; they do not prevent and/or suppress epileptogenesis—a process that converts a healthy brain into an epileptic one after the occurrence of the damage factor, i.e., infection, stroke, or trauma [7]. Thirdly, the occurrence of burdensome side effects related to the anticonvulsant medication results in the discontinuation of pharmacotherapy in up to 25% of patients and the inability to achieve fully effective therapeutic doses [8]. Considering the above-described problems, the coexistence of psychological and psychiatric disorders causes additional complications in the therapy of patients with epilepsy. There is still an urgent need to discover new medications that are safe and effective in the treatment of epilepsy as well as comorbid disorders.

**Figure 1:**
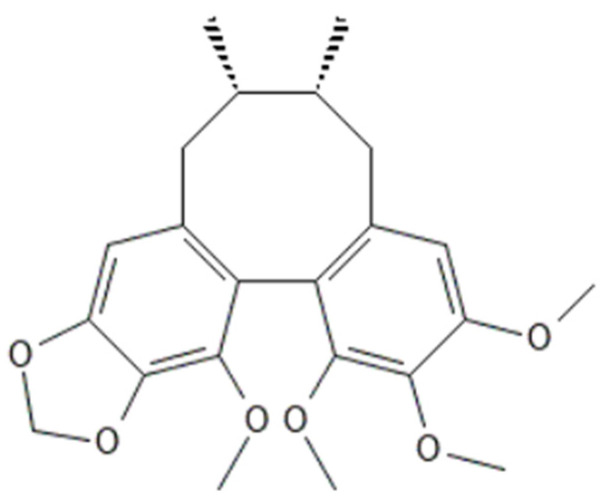
The chemical structure of schisandrin B.

One strategy to find new anticonvulsant medications and improve the therapy of epilepsy is to screen natural compounds from plants used in folk medicine [9,10]. In the present study, we investigate the potential anticonvulsant properties of schisandrin B using zebrafish larvae and mouse pentyleneterazole (PTZ) seizure tests. Schisandrin B (Figure 1) is one of the main active compounds of *Schisandra chinensis*—a traditional Chinese herb used for thousands of years to treat some neurobehavioral disorders, including insomnia and dysphoria [11,12]. The experimental studies confirmed the previously known traditional curative properties of *Schisandra chinensis* extracts [12,13] and demonstrated other biological activities, i.e., anti-inflammatory, antioxidant, and antitumor properties [14], and possibilities for the medicinal use of this plant and its constituents in the treatment of some neurodegenerative diseases. Extracts of *Schisandra chinensis* fruits improved cognition and learning abilities, prevented some pathological changes in the brain of mice with an intracerebroventricular injection of amyloid-β_1–42_ (Aβ_1–42_) [15,16]. Li et al. [17] revealed that a lower generation of Aβ in schisandrin B-treated mice might result from a decreased transcription and translation of β-secretase 1—an enzyme responsible for the generation of Aβ peptides. Moreover, schisandrin B prevented scopolamine-induced dementia in mice by decreasing acetylcholinoesterase activity and increasing the acetylcholine level in the brain [18] and might also alleviate cognitive dysfunctions occurring during menopause [19].

An experimental study confirmed the antidepressant and anxiolytic properties of *Schisandra chinensis* [20]. The antidepressant-like activity of schisandrin B alone was associated with the enhancement of serotoninergic neurotransmission (i.e., increased serum serotonin concentration, mRNA expression of serotonin receptor, and brain-derived neurotrophic factor (BDNF)) as well as the attenuation of neuroinflammatory processes (i.e., decreased indoleamine-2,3-dioxygenase expression, microglia activation, and inflammatory cytokines, TNF-α, IL-1β, and IL-6, in the brain) [21]. Schisandrin B alleviated anxiety-like behavior in mice subjected to the acute stressor of forced swimming and this action partly occurred due to its antioxidant activity [22].

The main aim of the present study was to evaluate the influence of schisandrin B on convulsant activity in the PTZ-induced seizure tests in larval zebrafish and mice. An analysis of the local field potential (LFP) recordings from the optic tectum (midbrain), as well as *c-fos* and *bdnf* mRNA expressions, were used to verify the anticonvulsant effect previously observed in behavioral seizure tests in zebrafish larvae. This was followed by the evaluation of schisandrin B safety in zebrafish embryos and larvae analyzing their development and behavior in the light–dark transition and thigmotaxis tests. The employed models are recommended for the initial screening of potentially active compounds in the preclinical studies [23,24]. In silico studies were performed before studies involving mouse models to assess the properties of schisandrin B, particularly its ability to cross the blood–brain barrier (BBB). The potential of schisandrin B to pass the BBB was also confirmed based on the determination of changes in its brain concentration at eight different time points after intraperitoneal (*ip*) administration in a tailored HPLC-ESI-QTOF-MS/MS method. In addition, changes in the plasma level of schisandrin B were also evaluated. The intravenous (*iv*) PTZ test was employed to assess its anticonvulsant effect in mice. In the final stage of the study, molecular modeling was conducted to find potential targets for schisandrin B that may be involved in its pharmacological effects.

## 2. Results

### 2.1. Effect of Schisandrin B in the PTZ Seizure Tests in Zebrafish Larvae

First, we determined maximal tolerated concentration (MTC) of schisandrin B. Four-day-old fish were exposed for 24 h to the following concentrations of the compound, i.e., 0.75, 1.5, 3, 6, and 12 µM. After this time, each fish was scored to determine abnormalities, e.g., heart oedema, yolk sac abnormalities, lack of swim bladder, body curvature, etc., or death [25]. Additionally, we assessed the touch response to evaluate muscle performance and function [26]. All larvae incubated with schisandrin B at 12 µM died. Evident morphological changes were seen in larvae incubated in a solution of 6 µM. Neither obvious morphological changes nor touch response disturbance were noted in larvae incubated in solutions of schisandrin B at concentrations up to 3 µM. Considering this result, the dose of 3 µM was selected as MTC for the PTZ-induced seizure assays.

**Figure 2:**
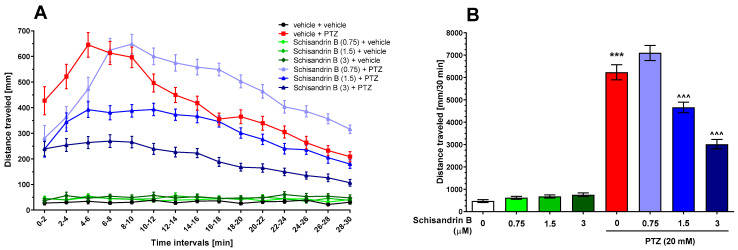
The effect of schisandrin B (0.75–3 µM) on PTZ-induced seizure-like activity in zebrafish larvae. The data are expressed as distance traveled (in mm) by larvae in 2 min-long time bins (**A**); total distance covered by larvae during 30 min of the assay (**B**). Data are analyzed using two-way or one-way ANOVAs with Bonferroni’s or Tukey’s post hoc tests, respectively. Data are presented as mean ± standard error of the mean (SEM). Group schisandrin B 3 µM + vehicle consisted of 36 zebrafish larvae, group schisandrin B 0.75 µM + PTZ consisted of 46 larvae, and the remaining experimental groups had 48 zebrafish larvae. *** *p* < 0.001 vs. vehicle + vehicle-treated group; ^^^^^ *p* < 0.001 vs. vehicle + PTZ-treated group. PTZ, pentylenetetrazole.

In our study, the influence of schisandrin B at concentrations of 0.75, 1.5, and 3 µM after 24 h of incubation on the zebrafish larvae convulsant behavior was evaluated. Two-way repeated measure analysis of variance (ANOVA) revealed statistically significant differences in the locomotor activity of larvae in the studied groups at different time points (treatment: F(7, 362) = 166.5, *p* < 0.001; time: F(14, 1907) = 53.97, *p* < 0.001; treatment × time interaction: F(98, 5068) = 13.08, *p* < 0.001; *n* = 36–48 per group, Figure 2A). The total distance traveled was analyzed with one-way ANOVA and statistically significant differences between the experimental groups were also revealed (F(7, 362) = 166.5, *p* < 0.001; *n* = 36–48 per group; Figure 2B). The lowest tested concentration of schisandrin B, i.e., 0.75 µM, did not exert anticonvulsant activity in PTZ-treated larvae but shifted the peak of PTZ-induced hyperlocomotion 4 min later. This meant that the full convulsant activity of PTZ developed 4 min later in schisandrin B-pretreated larvae than in the control (vehicle + PTZ-treated) group (*p* > 0.05; Figure 2A). The locomotor activity values of fish pretreated with 1.5 or 3 µM schisandrin B were 25.3% and 51.6% lower (both *p* < 0.001; Figure 2B), respectively, compared to the control (vehicle + PTZ-treated) group. None of the tested schisandrin B concentrations affected the basic locomotion of larvae (*p* > 0.05, Figure 2).

### 2.2. The Influence of Schisandrin B on PTZ-Induced LFP Discharges in Zebrafish Larvae

**Figure 3:**
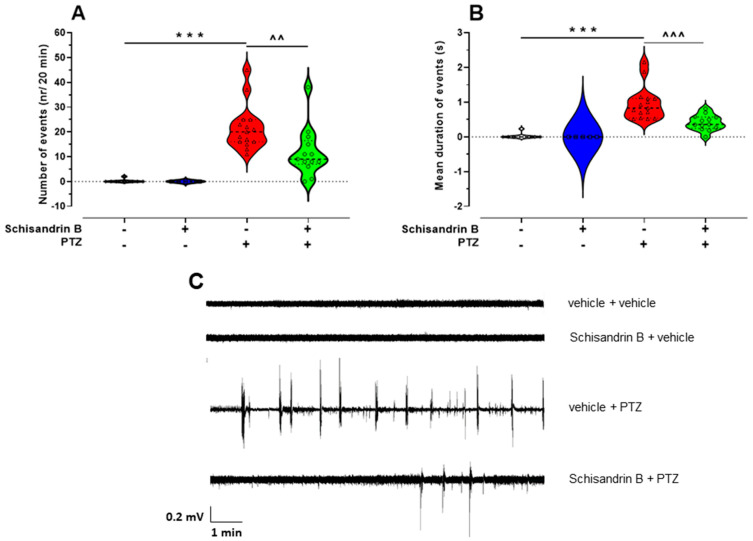
The effect of schisandrin B on epileptiform-like activity in the optic tectum of larval zebrafish treated with PTZ. Six-day-old fish were incubated with schisandrin B (3 µM) for 24 h and subsequently exposed to PTZ (20 mM). After a 5 min delay, LFP recordings from the larval optic tectum were obtained. Data are shown as: number of events (nr/20 min) (**A**); mean duration of events (s/20 min) (**B**); and representative LFP recordings (**C**). Data were analyzed using one-way ANOVA followed by Tukey’s post hoc test. Data are depicted as individual measurements (as symbols) and mean values. The experimental groups consisted of the following number of zebrafish larvae: vehicle + vehicle − 9, vehicle + PTZ − 15, schisandrin B + vehicle − 5, and schisandrin B + PTZ − 13. *** *p* < 0.001 vs. vehicle + vehicle; ^^^^^ *p* < 0.001, ^^^^ *p* < 0.01 vs. vehicle + PTZ. PTZ, pentylenetetrazole.

To verify behavioral outcomes from the zebrafish larval PTZ assay, LFPs from the larval optic tectum (midbrain) were recorded in 7-day-old larvae. One-way ANOVA showed statistically significant differences among the experimental groups in relation to both the number of epileptiform-like events (F(3, 38) = 18.48, *p* < 0.001; *n* = 5–15/group; Figure 3A) and their mean duration (F(3, 38) = 21.20, *p* < 0.0001, *n* = 5–15/group; Figure 3B). Schisandrin B significantly reduced the number of epileptiform-like events (*p* < 0.01; Figure 3A) as well as their mean duration (*p* < 0.001; Figure 3B) in comparison to the control (vehicle + PTZ-treated) group. Treatment with schisandrin B alone did not influence the brain activity of larval zebrafish (*p* > 0.05; Figure 3A,B), similar to the behavioral observations. Representative LFP recordings from experimental groups are presented in Figure 3C.

### 2.3. The Effect of Schisandrin B on c-fos and bdnf mRNA Expressions in PTZ-Treated Zebrafish Larvae

**Figure 4:**
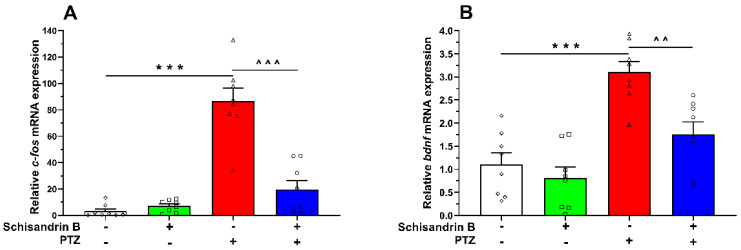
The effect of schisandrin B on *c-fos* (**A**) and *bdnf* (**B**) mRNA expressions in PTZ-bathed larval zebrafish. Six-day-old fish were incubated with schisandrin B (3 µM) for 24 h and subsequently exposed to PTZ (20 mM). After a 90 min delay, fish were collected and pooled (10/sample). Data were analyzed using one-way ANOVA with the Tukey’s post hoc test. Data are depicted as individual measurements (as symbols) and mean + SEM (*n* = 8/group). *** *p* < 0.001 vs. vehicle + vehicle; ^^^^^ *p* < 0.001, ^^^^ *p* < 0.01 vs. vehicle + PTZ. PTZ − pentylenetetrazole.

To assess the influence of schisandrin B on *c-fos* and *bdnf* mRNA expressions, zebrafish larvae were pretreated with schisandrin B (3 µM, 24 h) and incubated with PTZ (20 mM, 90 min); then, the larvae were pooled at 10/sample. One-way ANOVA of RT-qPCR data indicated that schisandrin B significantly affected both *c-fos* (F(3, 28) = 40.88, *p* < 0.001) and *bdnf* mRNA expressions (F(3, 28) = 17.30, *p* < 0.001) in comparison to the control (vehicle + PTZ-treated) group. PTZ application upregulated the expression of both genes (*p* < 0.001), while schisandrin B significantly attenuated these changes (*p* < 0.001 for *c-fos* and *p* < 0.01 for *bdnf*). Moreover, schisandrin B alone did not induce any changes in the mRNA expression of those markers (*p* > 0.05) compared to the respective control group, i.e., vehicle + vehicle-bathed fish (Figure 4).

### 2.4. Effect of Schisandrin B on Developing Zebrafish

**Figure 5:**
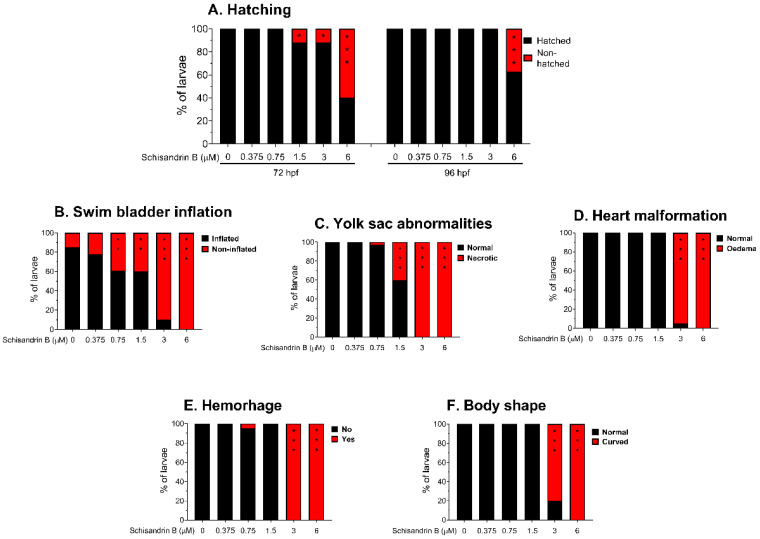
The effect of schisandrin B on hatching at 72 and 96 hpf (**A**); swim bladder inflation (**B**); yolk sac abnormalities (**C**); heart malformation (**D**); hemorrhage (**E**) and body shape of 96 hpf old larvae (**F**). Zebrafish larvae were exposed to schisandrin B at a concentration range of 0.375–6 µM for 95 h. All scores were performed in triplicates and the results were pooled together. The experimental groups consisted of the following number of zebrafish larvae: vehicle + vehicle − 36, schisandrin B 0.375 µM − 32, schisandrin B 0.75 µM − 33, schisandrin B 1.5 µM − 36, schisandrin B 3 µM − 36, schisandrin B 6 µM − 34. * *p* < 0.05; ** *p* < 0.01; *** *p* < 0.001 vs. control group, Fisher’s exact test.

In the first step of the toxicity study, we counted the number of embryos hatched at 72 and 96 h post-fertilization (hpf) in order to test whether schisandrin B caused any disturbances in zebrafish development. We noted that schisandrin B at concentrations of 1.5 and 3 µM delayed hatching in 72 h old embryos (both *p* < 0.05 vs. the control group); however, there was no difference between these groups and control littermates at 96 hpf (*p* > 0.05, Figure 5A). Moreover, 60% and 40% of larval zebrafish exposed to 6 µM of schisandrin B were delayed in hatching at 72 and 96 hpf, respectively (both *p* < 0.001; Figure 5A).

**Figure 6:**
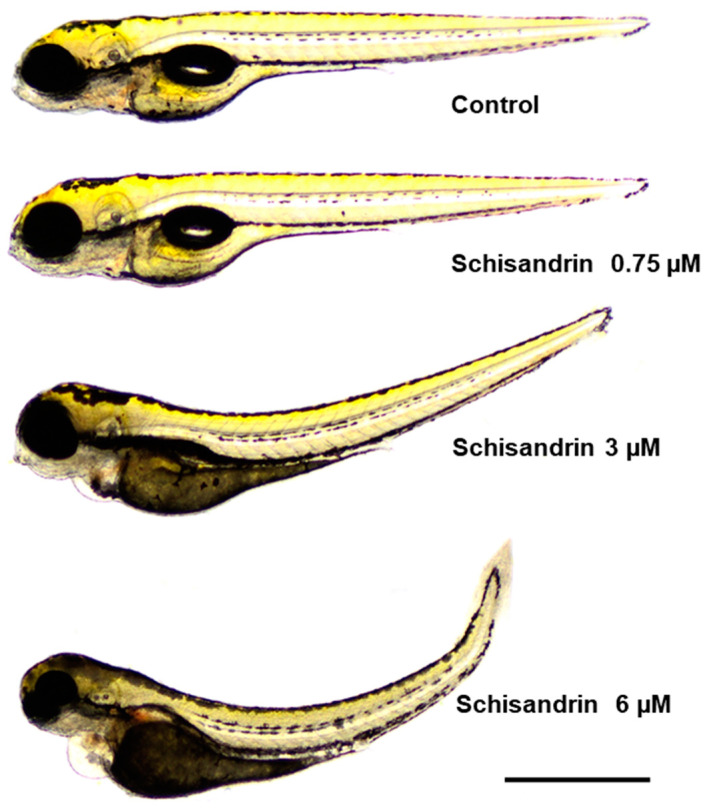
Representative images of the zebrafish larvae from the toxicity test. One hpf zebrafish embryo was exposed to different concentrations of schisandrin B (from 0.375 to 6 µM) for 95 h and, afterwards, pictures of the representative zebrafish larvae were taken. Larvae are shown to the same scale (bar = 1 mm).

Schisandrin B in a dose-dependent manner induced severe morphological abnormalities in developing zebrafish after 95 h of incubation. Only fish exposed to 0.375 µM of schisandrin B did not differ from their control counterparts (*p* > 0.05; Figure 5B–F). For doses of 0.75 and 1.5 µM, 40% of fish did not develop their swim bladders (*p* < 0.01 vs. the control group). Moreover, a concentration of 3 µM caused yolk sac abnormalities in 40% of fish incubated (*p* < 0.001). All or almost all zebrafish exposed to the highest doses of schisandrin B, i.e., 3 and 6 µM, experienced severe abnormalities—they did not inflate their swim bladders (*p* < 0.001) and yolk sac abnormalities (*p* < 0.001), heart oedema (*p* < 0.001), and hemorrhaging around the liver (*p* < 0.001) were observed. Moreover, fish bodies were curved (*p* < 0.001) and hyperpigmented at 96 hpf. For representative images, see Figure 6.

### 2.5. Effect of Schisandrin B in the Light–Dark Test in Zebrafish Larvae

**Figure 7:**
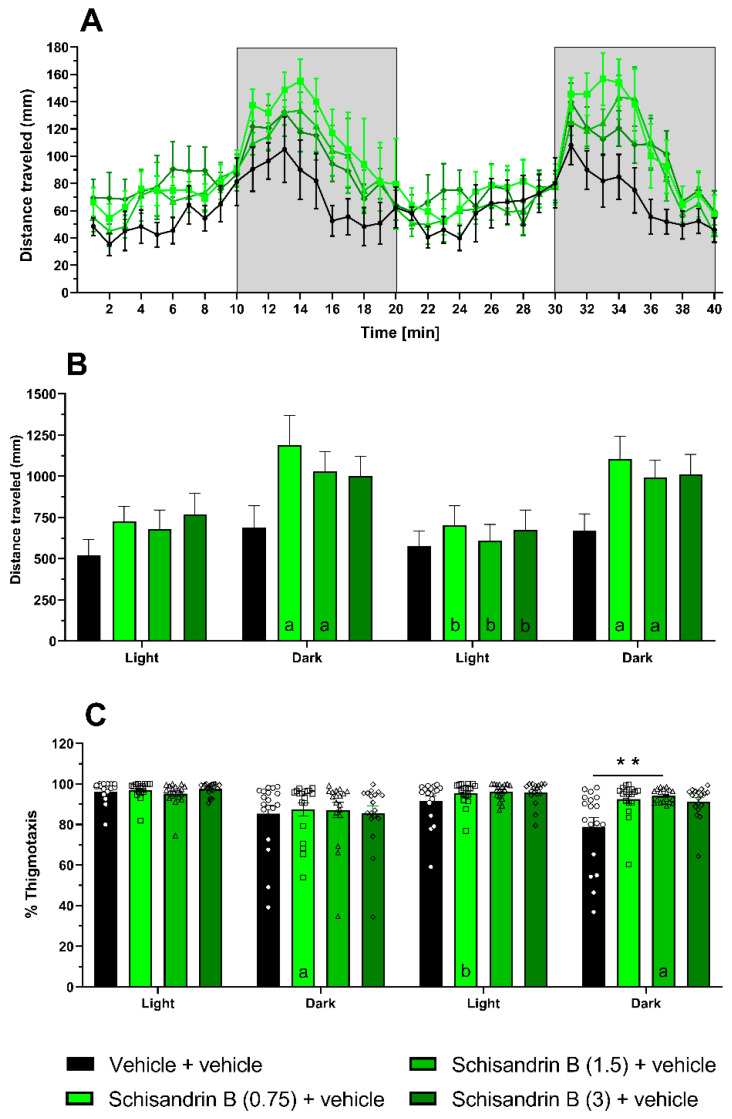
The effect of schisandrin B (3 µM) on zebrafish larval behavior in the light–dark and thigmotaxis tests. The data are shown as: distance traveled (in mm) by larvae when exposed to alternating light (100%) and dark (0%) conditions in 1 min-long time bins (**A**), aggregate data over the entire stimulus duration (10 min) (**B**), the percentage of time that the larvae are located in the outer zone of the swimming area when exposed to alternating light (100%) and dark (0%) conditions (**C**). Data were analyzed using two-way repeated measures with Tukey’s post hoc test. Data are presented as individual measurements (as symbols) and mean ± SEM. All experimental groups consisted of 18 zebrafish larvae. Letters indicate significant differences within treatment groups: a. comparison from light to dark phase, b. comparison from dark to light phase; ** *p* < 0.01.

The effects of schisandrin B on larval behavior in the light–dark test were represented in over 1 min time bins to reveal the temporal dynamics within each phase (Figure 7A) and the aggregate behavior per phase (Figure 7B). Two-way repeated measures of ANOVA revealed statistically significant differences in the distance traveled by larvae among the different treatment groups and illumination conditions (treatment: F(3, 68) = 1.722, *p* > 0.05; illumination condition: F(2.206, 150) = 29.58, *p* < 0.001; treatment × illumination condition: F(9, 204) = 1.547, *p* > 0.05; *n* = 18 larvae per group). During the dark phase, there was a tendency for all groups to travel a greater distance compared to the light phase, reaching statistical significance in most instances. The schisandrin B-treated groups, 0.75 (*p* < 0.01) and 1.5 µM (*p* < 0.05), consistently showed alternating reductions and increasing activity in the light and dark conditions, respectively. However, the treatment groups did not differ significantly during each illumination condition.

We also observed that the outer-zone preference of larvae was significantly influenced by both illumination conditions and schisandrin B treatment (two-way repeated measures ANOVA, treatment: F(3, 68) = 3.417, *p* < 0.05; illumination condition: F(2.067, 140.6) = 15.05, *p* < 0.001; treatment × illumination condition: F(9, 204) = 1.766, *p* > 0.05; *n* = 18 per group; Figure 7C). Larvae across all groups spent more time in the outer zone during the light phase than in the dark phase, and thigmotaxis in the light phase was generally higher than in the dark phase. Although there were no significant differences in the zone preference between the groups in most cases, we observed a tendency towards statistical significance in the schisandrin B-treated groups compared to the vehicle-treated group during the second dark phase (vehicle-treated group vs. schisandrin B 1.5 µM-treated group, *p* < 0.01; vs. schisandrin B 0.75 µM-treated group, *p* = 0.0506 and vs. schisandrin B 3 µM-treated group, *p* = 0.0758).

### 2.6. Analysis of BBB Descriptors

To observe if schisandrin B could effectively cross the BBB, some pharmacokinetic parameters were calculated using ACD/Percepta software (version 2012, Advanced Chemistry Development, Inc., Toronto, ON, Canada). The following parameters were calculated: logBB—the distribution of a substance in the blood–brain area (the BBB penetration descriptor), logPS—the rate of passive diffusion/permeability (the permeability–surface area product), logPS*_fu,brain_—the brain/plasma equilibration rate, Fu—the fraction unbound in plasma, and Fb—the fraction unbound in the brain. The results are presented in Table 1.

In further studies, other physicochemical parameters, i.e., steric, electronic, and lipophilic ones, were calculated. The following logarithms of partition coefficients, as descriptors of lipophilicity, were calculated: n-octanol/water (logPow) and cyclohexane/water (logPcw). The results are presented in Table 2.

Moreover, according to the linear free-energy relationship methodology [27,28], the excess molar refraction (E) was determined. This parameter combined with the lipophilic descriptor logPow and the hydrogen-bonding parameter ΔlogP (the difference between the logarithms of n-octanol/water and cyclohexane/water partition coefficient values) provided a promising combination to estimate the logBB values [29].

To calculate the logBB value for schisandrin B, previously established in [29], the quantitative structure–activity relationship (QSAR) model was also used (Equation (1)). The model was set up to examine the quantitative relationship between the ability of a compound to cross the BBB and the molecule structure.

 logBB = −0.114 − 0.098 ΔlogP + 0.278 logPow + 0.218E(1)

In the equation presented above, *n* = 40, R^2^ CV = 78.25%, R^2^ pred = 74.02%, and S = 0.436.

Immobilized artificial membrane (IAM) liquid chromatography was also used as the biomimetic system to determine the BBB permeability of schisandrin B. For this purpose, the value of the logarithm of the retention factor extrapolated to pure water (logkw) was determined. The obtained logkw value was used for the calculation of the logBB-IAM value based on the above-mentioned QSAR equation, instead of the logPow parameter.

**Figure 8:**
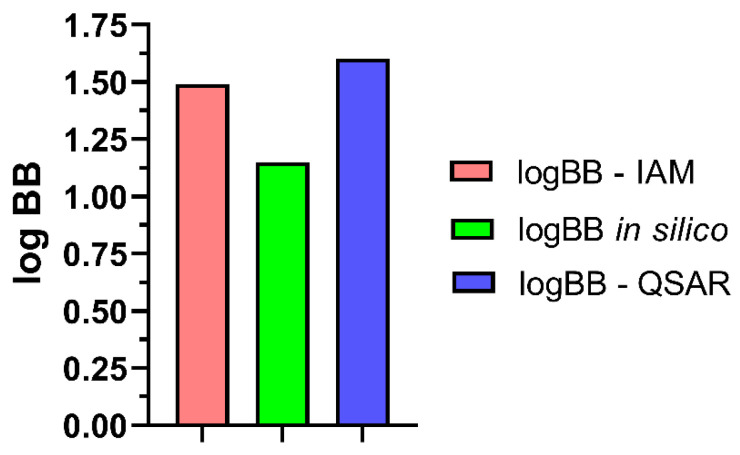
LogBB values calculated for schisandrin B. logBB in silico—the value calculated using ACD/Percepta software, logBB-QSAR—the value calculated based on Equation (1), and logBB-IAM—the value determined from the biomimetic IAM system.

The obtained logBB values are presented in Figure 8.

### 2.7. Changes in Schisandrin B Concentration in Mouse Serum and Brain Tissue

**Figure 9:**
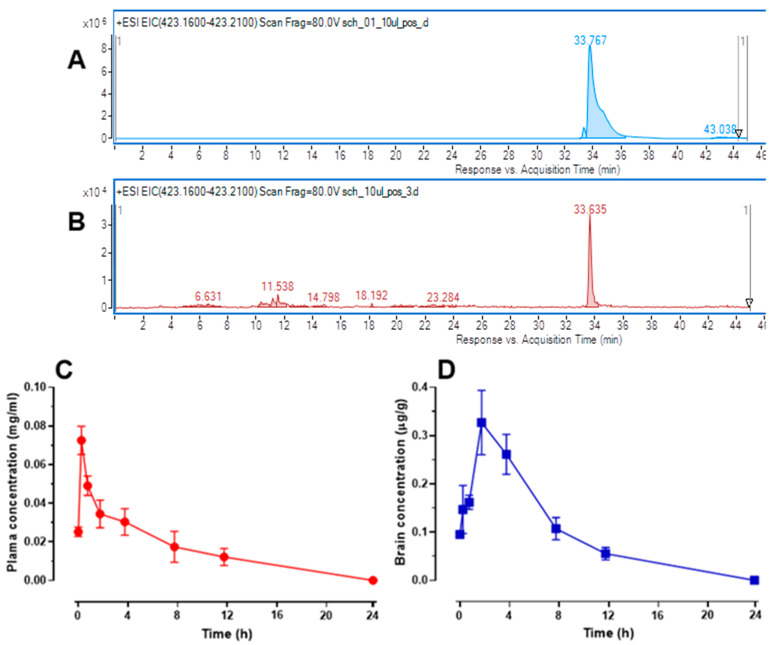
Mass chromatogram recorded in the positive ionization mode for the reference solution of schisandrin B (concentration 1 mg/mL, injection volume: 10 µL) (**A**); extracted ion chromatogram of schisandrin B in brain tissue recorded in the positive ionization mode (**B**), and changes in schisandrin B concentration in the blood plasma (**C**) and brain tissue (**D**) at 8 time points, i.e., 15 and 30 min as well as 1, 2, 4, 8, 12, and 24 h, after its *ip* administration at a dose of 50 mg/kg in mice. Plasma and brain concentrations of schisandrin B are presented as mean ± standard deviation (SD) (*n* = 6).

The applied chromatographic protocol was tailored to elevate the sensitivity of the mass spectrometer toward the detection of schisandrin B in biological samples. For this purpose, the settings of gas and sheath gas temperatures, gas flow rate, capillary voltage, fragmentation voltage, and collision energies were optimized using the solution of schisandrin B standard (1 mg/mL). As a result, the capillary voltage was set at 3000 V (that was selected from the range of 2800–4000 V), gas and sheath gas temperatures were 275 and 300 °C (from the range of 250–375 °C), gas flow was 12 L/min (from the range of 8–12 l/min), and the fragmentation was 80 V (from the range of 70–130 V). In the analysis, the authors were able to detect the peak of schisandrin B, which was visible in the mass chromatogram as a sodium adduct [M + Na]^+^ at the same retention time as the reference compound (Figure 9A,B) and with the same m/z parameter. The accurate mass measurement of the molecule and the analysis of its MS/MS spectra provided sufficient information for the identification of this natural product in comparison to the reference compound. Schisandrin B in the tested samples was detected with m/z of 423.1793 Da. The measurement showed a 2.17 ppm deviation from the theoretical mass. The molecule was characterized by the double-bond equivalent value (DBE) of 13.

The quantitative analysis of the compound of interest in the biological samples was performed based on the calibration curve equation that was obtained for the standard of schisandrin B (purity > 95%, Glentham Life Sciences Ltd., Corsham, UK) that was injected in the same way as the tested samples in the range of concentrations from 0.0005 to 0.1 mg/mL.

Fifteen min after *ip* administration of schisandrin B at a dose of 50 mg/kg, it was detected in the serum at a concentration of 0.025 ± 0.002 µg/mL and in the following time point, i.e., 30 min after administration, its level increased to the highest noted value of 0.073 ± 0.007 µg/mL. At later time points, the concentration of schisandrin B in plasma was gradually reduced; although, even 8 and 12 h after administration, the level of schisandrin B in the blood plasma was quite high and reached 0.017 ± 0.008 µg/mL and 0.012 ± 0.004 µg/mL, respectively. We did not observe any peak of the compound in the plasma 24 h after the injection. Levels of schisandrin B in mouse plasma are presented in Figure 9C.

A detectable concentration of schisandrin B in the brain tissue was found as early as 15 min after its administration and reached 0.095 ± 0.003 µg/g. The level of this compound increased at subsequent time points (i.e., 30 and 60 min after administration) and reached a maximum value of 0.327 ± 0.027 µg/g 2 h after its administration. Schisandrin B level gradually decreased at the next time points (i.e., 4, 8, and 12 h) and it was not detected in the samples collected 24 h after its administration. Changes in schisandrin B concentration in the brain tissue are presented in Figure 9D.

### 2.8. Effect of Schisandrin B on Seizure Thresholds in the iv PTZ Seizure Test in Mice

**Figure 10:**
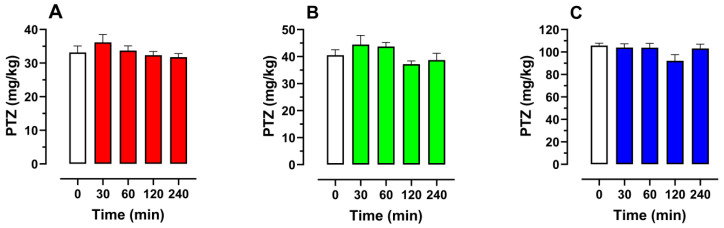
The time–course effect of schisandrin B (50 mg/kg, *ip*) on the threshold for myoclonic twitches (**A**), generalized clonic seizures with the loss of righting reflex (**B**), and tonic seizures (**C**) in the *iv* PTZ test in mice. Control group and groups tested 30 and 240 min after schisandrin B treatment consisted of 11 mice while groups tested 60 and 120 min after schisandrin B treatment had 12 animals. Data were analyzed using one-way ANOVA with the Tukey’s post hoc test. Data are depicted as mean ± SEM. White bars represent the control groups while colored represent schisandrin B-treated groups.

Based on the data concerning changes in schisandrin B levels in the brain tissue, we decided to evaluate its anticonvulsant effect (previously noted in the zebrafish larvae PTZ assay) in the timed PTZ test in mice at 4 different time points, i.e., 30, 60, 120, and 240 min, after its *ip* administration at a dose of 50 mg/kg. There were no statistically significant changes in the seizure thresholds for the myoclonic twitches (one-way ANOVA: F(4, 50) = 1.026; *p* > 0.05), generalized clonic seizures (one-way ANOVA: F(4, 50) = 2.16, *p* > 0.05), as well as tonic convulsions (one-way ANOVA: F(4, 50) = 2.005, *p* > 0.05) in mice (Figure 10A–C).

### 2.9. Molecular Docking

In order to rationalize, at the molecular level, the observed in vivo anticonvulsant activity of schisandrin B in zebrafish larvae, molecular modeling was performed. As PASS 2022 and Pharma Expert 2022 (geneXplain, Wolfenbüttel, Germany) software could not able indicate the possible molecular targets for the observed activity of schisandrin B (which confirmed the originality of its structure among potential ASMs), the respective receptors were selected based on our knowledge and the available literature. The activation of dopamine D_2_ or the serotonin 5-HT_2A_ receptor, the blockade of the NMDA ion channel, and binding in the benzodiazepine (BDZ) site of the GABA_A_ receptor were studied as the possible molecular mechanisms of the anticonvulsant activity of schisandrin B.

**Figure 11:**
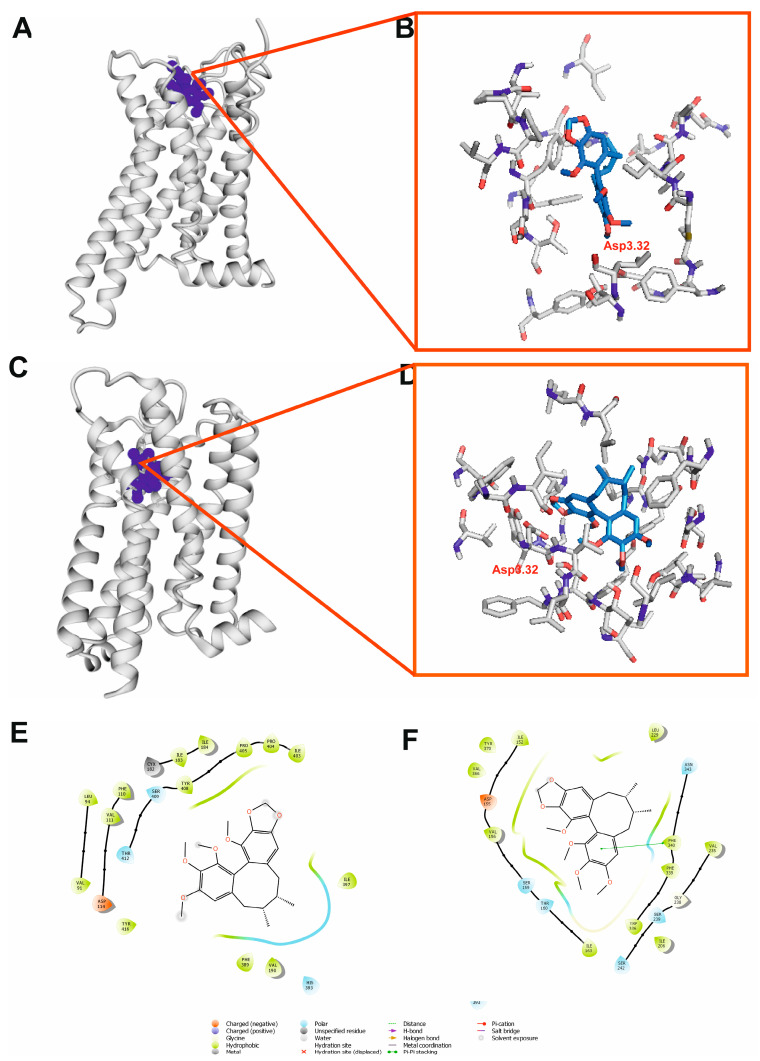
Molecular interactions of schisandrin B with D_2_ receptor (**A**,**B**,**E**) and 5-HT_2A_ receptor (**C**,**D**,**F**) based on molecular docking. (**A**,**C**) Overview of ligand pose in the receptor. Receptor shown in gray in cartoon representation, ligand shown as blue balls; (**B**,**D**) 3D details of the binding site. The most important protein residues are shown as sticks with gray carbon atoms. Ligand shown as sticks with blue carbon atoms; (**E**,**F**) 2D overview of the binding site.

The results of the molecular docking of schisandrin B to the orthosteric pockets of D_2_ and 5-HT_2A_ (both are G-protein-coupled receptors—GPCR) receptors are presented in Figure 11. The most striking difference between the positions of schisandrin B in the dopamine D_2_ receptor (Figure 11A) and serotonin 5-HT_2A_ receptor (Figure 11C) is the shallow position of the ligand in the D_2_ receptor. Although the molecular docking was performed on the orthosteric site, in the case of the D_2_ receptor, schisandrin B was not able to fully penetrate the orthosteric pocket and its position in the receptor could be classified as allosteric (Figure 11B,E). As for the 5-HT_2A_ receptor, schisandrin B occupied the classical orthosteric pocket (Figure 11D,F). For both receptors, the docking of schisandrin B resulted in favorable values for the Glide score (−5.341 and −4.775, respectively) and MMGBSA dG bind energy (−50.27 and −33.54 kcal/mol, respectively) (Table 3).

**Figure 12:**
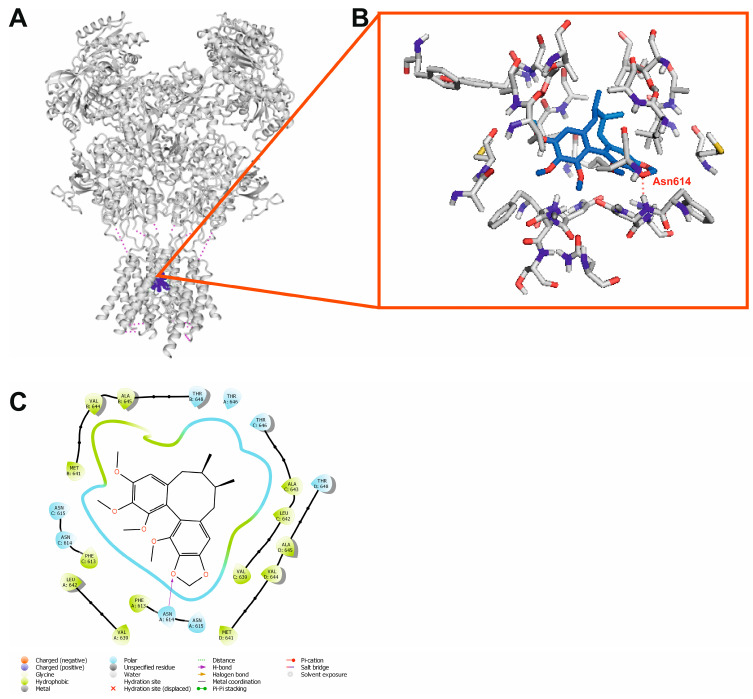
Molecular interactions of schisandrin B with NMDA receptor based on molecular docking. (**A**) Overview of ligand pose in the receptor. Receptor shown in gray in cartoon representation, ligand shown as blue balls; (**B**) 3D details of the binding site. The most important protein residues are shown as sticks with gray carbon atoms. Ligand shown as sticks with blue carbon atoms. Polar interactions shown as red dashed lines; (**C**) 2D overview of the binding site.

Our analysis revealed that schisandrin B interacted with the NMDA ion channel (Figure 12B,C) by blocking it and acting as an uncompetitive antagonist. One of its oxygen atoms formed a hydrogen bond with Asn614. Other ligand–receptor interactions were mainly of a hydrophobic nature. The Glide docking score and MMGBSA dG bind energy were also favorable for this ligand–receptor complex (−7.125 and −42.63 kcal/mol, respectively, Table 3).

**Figure 13:**
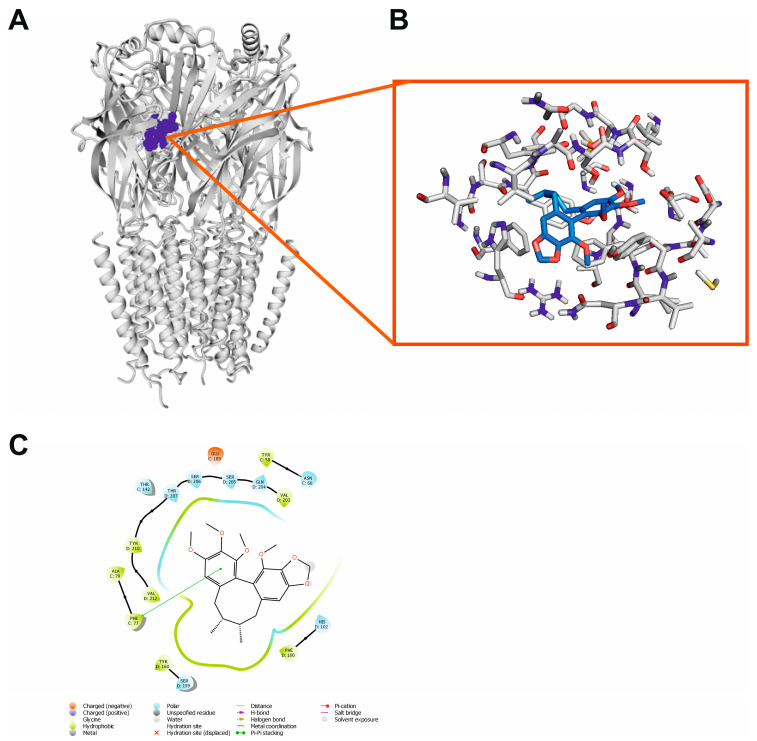
Molecular interactions of schisandrin B with GABA_A_ receptor based on molecular docking. (**A**) Overview of ligand pose in the receptor. Receptor shown in gray in cartoon representation, ligand shown as blue balls; (**B**) 3D details of the binding site; (**C**) 2D overview of the binding site.

Finally, the anticonvulsant activity of schizandrin B may be rationalized by its interactions with the GABA_A_ receptor (Figure 13A). We showed here that schisandrin B could interact with the BDZ site of the GABA_A_ receptor, similarly to alprazolam. In this case, there hydrophobic-only interactions were observed, in particular Phe77 was involved π–π stacking contact with one of the aromatic rings of the ligand (Figure 13B,C). Glide docking score and MMGBSA dG bind energy were also favorable for this ligand–receptor complex (−6.955 and −49.25 kcal/mol, respectively, Table 3) indicating the possibility of attractive interactions.

## 3. Discussion

One of the main goals of the present study was to evaluate the anticonvulsant properties of schisandrin B. The main purpose of anticonvulsant drugs is to control convulsions based on the attenuation of excessive excitatory processes in the central nervous system [30]. Natural products can be treated similar to an adjuvant or even, in certain cases, a primary treatment for various kinds of diseases. Some of the plant metabolites could be interesting sources of compounds with anticonvulsant properties, e.g., *Melissa parviflora* [31], *Berberis sibrica* [32], *Zingiber officinale* [25], or *Carissa edulis* [33].

Schisandrin B is one of the compounds that are the most abundant in *Schisandra chinensis*. It is a secondary metabolite that belongs to the class of organic compounds named hydrolysable tannins. It has proven, among others, anti-inflammatory [34], antioxidant [22,35], cardioprotective [36], liver-protective [37], as well as neuroprotective [38,39] properties. Wei et al. [40] demonstrated that *Schisandra chinensis* extract exhibits sedative–hypnotic effects in rats and affects levels of some neurotransmitters (i.e., glutamate, GABA, noradrenaline, dopamine, and serotonin) in the brain. Moreover, the sedative and hypnotic activities of schisandrin B in rodents were previously revealed by Li et al. [41]. This effect results from the modulation of GABAergic and glutamatergic neurotransmission systems, more precisely, from an increased GABA/glutamate ratio both in the brain structures and peripheral blood, as well as the upregulated mRNA expression of α_1_ and γ_2_ subunits of the GABA_A_ receptor in the brain tissues [41]. Described mechanisms of action suggest that schisandrin B might also present anticonvulsant activity. We verified this hypothesis in our present study firstly using the PTZ-induced seizure assay in larval zebrafish. Zebrafish have been used for many years in biomedical studies to model various neurological and psychiatric disorders and, at the present time, they are recommended as a respective animal model for the preclinical screening of drugs with central nervous system effects [42,43].

Our results clearly show the anticonvulsant effect of schisandrin B that reduces behavioral (i.e., distance traveled) and electrographic (i.e., number and duration of epileptiform-like events) signs of seizures in PTZ-treated zebrafish larvae. Moreover, the studied compound influenced the mRNA expression of two molecular markers, i.e., *c-fos* and *bdnf*. C-Fos is a recognized indirect marker of the functional activity of neurons and numerous studies have proved its involvement in the seizure generation process both in rodent [44,45] and zebrafish [32,46] PTZ-induced seizure/epilepsy models. Similar to *c-fos*, epileptic insults also upregulate BDNF expression both at the mRNA and protein levels in brain structures [32,44,47]. BDNF influences, among others, the release of neurotransmitters, neuronal morphology, and synaptogenesis [48]. Moreover, BDNF binding to tropomyosin receptor kinase B (TrkB) activates various intracellular pathways and induces some transcription factors, including *c-fos*. Numerous studies demonstrated various and complex influences of BDNF on GABAergic neurotransmission. BDNF might enhance GABA transporter 1 activity and thus decrease presynaptic GABA concentration. Moreover, the expression of GABA_A_ receptor subunits might also be changed and GABA_A_ functions might be disrupted through an elevated Ca^2+^ intracellular concentration [49]. Considering that schisandrin B increases GABA levels and the expression of GABA_A_ receptor subunits [41], we can speculate that a decrease in the mRNA expression of *bdnf* in schisandrin B-treated zebrafish larvae can enhance GABA neurotransmission and thus reduce seizure activity in the PTZ seizure test. Moreover, a reduced *bdnf* expression might also be responsible, at least partially, for the decrease in *c-fos* expression in schisandrin B-treated zebrafish larvae.

In the context of anticonvulsant properties, it is crucial to study the ability of schisandrin B to cross the BBB and its distribution to the brain—the site of its pharmacological action. The BBB is a specialized barrier created by endothelial cells of the capillaries and glia in the brain that is essential for maintaining the homeostasis of the brain and ensuring its protection from potentially harmful substances; however, it is also a major challenge for drug delivery to the central nervous system [49]. Lipophilicity is one of the most crucial features influencing the ability of substances to cross biological barriers [50]. It strongly determines the ability of a chemical compound to be distributed in the biosystem. To observe if schisandrin B can effectively cross the BBB, some pharmacokinetic parameters were calculated using computational methods (Table 1).

The scientific reports indicated that the lower the values of the BBB permeability–surface area product (PS) or the fraction unbound in the brain (Fb), the longer the time it takes to reach brain equilibrium [51]. To achieve the desired pharmacological effect, it must be emphasized that drugs in the blood are present in the unbound form but may also be bound to plasma proteins and erythrocytes. The free drug hypothesis postulates that all the distribution processes depend on the unbound drug concentration [52]. In our experiment, it was shown that the values of both the fraction unbound in the brain and the fraction unbound in the plasma were very low (0.01 and 0.11, respectively). Moreover, the brain/plasma equilibration rate was relatively high (logPS_Fu,brain_ −3.2). A high rate of penetration results from the high permeability expressed as PS as well as low binding to the brain tissue. However, most contemporary biomimetic and computational methods estimate the ability of a molecule to cross the BBB based on the logBB parameter being the brain/blood partitioning one in a steady state. It is defined as the logarithmic ratio between the concentration of a substance in the brain and its concentration in the blood [53]. As a result of the analysis of all the above-mentioned pharmacokinetic parameters, it can be concluded that schisandrin B is able to cross the BBB.

However, it should be strongly emphasized that the permeability of the BBB is determined by the physicochemical properties of a compound, mainly by its lipophilicity as well as it is influence by the active transport at the BBB [52]. Lipophilic descriptors characterize the transport and resorptive properties of a compound, including the interaction of a molecule with competing binding sites and metabolizing enzymes. Lipophilicity is most often characterized by the substance partition coefficients in various experimental systems, since hydrophobic interactions are responsible for the interaction of a molecule with the molecular target. In our case, lipohilicity was determined by the following logarithms of partition coefficients: n-octanol/water (logPow) and cyclohexane/water (logPcw). Analyzing these parameters, it can be concluded that schisandrin B is a highly lipophilic substance, capable of crossing biological barriers, including the BBB. According to Hansch’s [50,54] approach, the permeability of a substance through biological barriers was also determined by the other physicochemical parameters of a molecule, including steric and electronic ones. Schisandrin B has a molar weight equal to 400.46 g/mol, and it is polarizable. Moreover, to penetrate the BBB and act on receptors in the central nervous system, the topological polar surface area (TPSA) should be less than 90 Å2 [55]. Our in silico studies showed that the TPSA value of schisandrin B was equal to 55.38 Å2. To summarize, the analysis of the physicochemical parameters performed, among others, based on the Hansch approach [50,54] and the Lipinski “rule of five” [56] proved that schisandrin B was capable of crossing the BBB.

The quantitative structure–activity relationship (QSAR) plays an important role in contemporary drug design. The QSAR methodology is based on the theory that biological activity is a sum of various interactions of a compound during its transport through biological barriers and in the reaction with the sites of action (receptor) [57]. In this experiment, QSAR studies were used for the investigation of the dependence between the structure of schisandrin B and its capability to cross the BBB expressed as logBB. Many QSAR models for the estimation of the ability of compounds to cross the BBB were previously established. Most of them were based on both the Hansch and LFER models [29,53,57,58,59,60,61]. To calculate the logBB values for schisandrin B, the QSAR model previously obtained was used (Equation (1)) [29]. This predictive model is based on the lipophilic properties (logPow), excess molar refraction (E) taken from the Abraham model, and the hydrogen-bonding potential, expressed as the difference between the logarithms of n-octanol/water and cyclohexane/water partition coefficient values (ΔlogP). The logBB-QSAR value was calculated based on the values obtained in silico (see Table 2). Similar values of logBB in silico and logBB-QSAR may suggest that the QSAR model is applicable and predictive in assessing the permeation of schisandrin B through the BBB. To confirm this assumption, biomimetic chromatographic studies were performed using the immobilized artificial membrane column (IAM) being a more realistic model than a theoretical one.

The use of IAMs as chromatographic packing materials started the era of biomimetic chromatographic partitioning systems [38]. The permeability of a substance through biological barriers is determined by the membrane partition coefficient, which, in turn, is difficult to measure in vivo [62]. The usefulness of IAM liquid chromatography has been extensively explored since the mid-nineties [63]. The IAM column contains a monolayer of phosphatidylcholine covalently bound to a propylamino-silica core. It is used in high-performance liquid chromatography (HPLC) systems with a physiological buffer as an eluent [64]. A monolayer of phosphatidylcholine immobilized on a silica support is an imitation of the phospholipid bilayer that can mimic drug/biomembrane interactions [65]. The retention on the IAM stationary phase is often related to the specific biological activity of a given compound, including permeation through the BBB, oral absorption, transdermal transport, etc. [63,66].

In this study, IAM chromatography was used to determine the BBB permeability of schisandrin B. For this purpose, the value of the logarithm of the retention factor extrapolated to pure water (logkw) was determined. It was recognized to be an alternative to the logPow lipophilicity descriptor [67]. Therefore, the obtained logkw value was used as a lipophilic descriptor for the calculation of logBB based on the QSAR model (Equation (1)). This value was then compared with the logBB in silico value and logBB-QSAR one. A comparison of these three logBB values calculated with different methods is shown in Figure 8. To date, the literature does not present such computational and biomimetic studies with regard to schisandrin B.

In our study, we decided to confirm the results from the zebrafish larvae PTZ seizure test (i.e., anticonvulsant activity of schisandrin B) and in silico analysis (i.e., the ability of schisandrin B to cross the BBB) in in vivo studies in mice. The increasing amount of data confirms that the BBB in zebrafish shares both structural and functional similarities with the mammalian BBB, and thus zebrafish are considered useful screening tools for the estimation of drug distribution through the BBB [68]. However, the development and maturation of the BBB in the zebrafish larvae occurred between 3 and 10 days post-fertilization (dpf) [69]. Since, in the PTZ seizure assay, 6 dpf zebrafish larvae were used, there was some probability that the distribution of schisandrin B into the brain may not have exactly correlated with that in adult zebrafish, mammals, and humans. In our study, changes in schisandrin B concentration in mouse brain tissues were determined to verify its ability to penetrate the BBB. Moreover, the determination of the time point of maximal concentration in the brain was also utilized to choose appropriate time points to evaluate the time–course effect of schisandrin B in the IV PTZ test in mice. For a more comprehensive view of the pharmacokinetic profile of schisandrin B, changes in its plasma concentration were also determined.

First, our results confirm the ability of schisandrin B to cross the BBB. Moreover, it is worth noting that the plasma concentration of schisandrin B reaches the highest peak much earlier, i.e., 30 min after its administration, in comparison with brain tissue content, which is the highest 120 min after injection. Our data also indicate that schisandrin B is distributed into the brain relatively rapidly (it is recognized in the brain tissue 15 min after administration) and is slowly eliminated (a measurable concentration of schisandrin B is persistent up to 12 h). A similar conclusion was previously presented by Zhu et al. [70]. Moreover, our data on the changes in schisandrin B concentration in the brain tissue are consistent with the results of the study conducted by Wang et al. [71] in which micronized schisandrin B was administered orally at a dose of 20 mg/kg in rats. The highest concentration of schisandrin B in the brain tissue was also observed 120 min after its administration; however, what was interesting was that its level was dependent on the sex of the animals—it was much higher in female than in male rats. Since in our study we used schisandrin B, which was not changed by any kind of formulation and only male mice were used in the PTZ test, there was some probability that using female animals and any kind of formulation that increased the bioavailability of the compound might have changed the distribution of schisandrin B and resulted from the seizure test in mice.

The anticonvulsant effect noted in the zebrafish larvae model was verified in the IV PTZ seizure test in mice. This model is a very sensitive test that allows us to evaluate the effect of the studied compounds on three separate components of the seizure behavior, i.e., myoclonic, generalized clonic, and tonic seizures [72]. Although our results confirm the ability of schisandrin B to penetrate the BBB, we did not note its anticonvulsant effect in the IV PTZ test in mice.

The ability of compounds to cross the BBB is a key condition in the development of new drugs influencing the central nervous system functions; however, their effectiveness is also determined by other factors, i.e., the degree of binding by brain tissue and affinity to the target. The high total brain concentration of the compound does not reflect the real amount that is available to reach the respective target in brain tissues, and thus it does not always result in the high efficacy of the drug [73,74]. Considering the results from our in silico analysis and the seizure tests on mice, we can suppose that the lack of an anticonvulsant effect might have resulted from a very low level of the unbound fraction of schisandrin B in the brain. Although this compound revealed a significant anticonvulsant effect in the zebrafish seizure assay, it did not alleviate convulsant activity in the mice because the level of the unbound fraction may not have been sufficient for an effective influence on the cellular targets, for example, GABA_A_ and/or glutamate receptors (two important targets for anticonvulsant compounds). Since we did not determine the unbound fraction of schisandrin B in the brain, further and more precise studies are necessary. Moreover, although Afrikanova et al. [75] validated the PTZ seizure assay in zebrafish larvae as a useful method that might be employed to screen compounds with potential anticonvulsant properties, they also noted some differences in the efficacy of the individual ASMs against PTZ-induced seizures in rodent and zebrafish larvae. These discrepancies might not only result from space, but also from the differences in the maturity of the brain as well as from the uptake and metabolism of the studied compounds [75].

To identify some possible targets for schisandrin B that were responsible or its anticonvulsant effect in the zebrafish larvae, we conducted an analysis of the molecular docking, taking into consideration the selected receptors potentially relevant for this effect. Dopamine receptor modulation affects susceptibility to seizures [76]. In particular, dopamine D_2_ receptor agonists are anticonvulsants and decrease pilocarpine and kindled seizures [77,78], while antagonists of this receptor are proconvulsant [79]. Similarly, the activation of the serotonin 5-HT_2A_ receptor may also have an antiseizure effect. The 5-HT_2A_ receptor negatively controls the expression of experimental absence seizures, and selective agonists of this GPCR can lead to the creation of innovative anti-absence drugs [80]. Molecular modeling was performed to investigate if schisandrin B could bind to these GPCRs in an active conformation to exert a possible antiseizure effect.

However, it must be emphasized that schisandrin B does not fulfill the classical pharmacophore model requirements for orthosteric ligands of aminergic GPCRs as it does not possess a protonatable nitrogen atom that can interact with the conserved Asp3.32. [81,82]. Thus, such an interaction was not observed and neither were any other polar interactions observed in the case of these two ligand–receptor complexes. There are, however, many ligands of aminergic GPCRs that either do not possess a protonatable nitrogen atom [83] or do not have a nitrogen atom at all, such as salvinorin A—a potent agonist of the κ opioid receptor [84] (also possessing aspartate in the 3.32 position). The lack of polar interactions in the ligand–receptor complexes does not need to result in weak binding only, as hydrophobic interactions are a promising driving force for biomedical applications [85]. Such interactions were observed in the case of complexes of schisandrin B with D_2_ and 5-HT_2A_ receptors. The allosteric binding pocket in the D2 receptor is formed i.a. by Phe389 (6.51), His393 (6.55), Tyr408 (7.34), Ile403 (el3), Pro404 (7.30), and Pro405 (7.31). The orthosteric binding pocket in 5-HT2A is constituted, i.e., by Asp155 (3.32), Phe339 (6.51), and Phe340 (6.52). Phe340 is involved in π–π stacking contact with one of the aromatic rings of the ligand. The favorable molecular docking results of schisandrin B to D_2_ and the 5-HT_2A_ receptor in active conformation indicate the agonism or positive allosteric modulation of these receptors by schisandrin B as the possible molecular mechanism of its antiseizure activity.

Another plausible explanation for the antiseizure activity of schisandrin B is its ability to block the NMDA ion channel (Figure 12A). It is well known that competitive and uncompetitive NMDA antagonists exert an anticonvulsant effect [86,87] as the activation of NMDA receptors induces oxidative stress in neurons and triggers excitotoxicity. Moreover, our analysis revealed that the manner in which schisandrin B interacted with the ion channel in the NMDA receptor was similar to that of phencyclidine—a non-competitive NMDA receptor antagonist with anticonvulsant properties [88].

Finally, the anticonvulsant activity of schisandrin B may also be rationalized by its interactions with the GABA_A_ receptor (Figure 13A). GABA, the main inhibitory neurotransmitter in the cerebral cortex, maintains the inhibitory tone that counterbalances neuronal excitation [89]. When this balance is perturbed, it may result in seizures. The results of our analysis reveal that schisandrin B might act as a positive allosteric modulator of the GABA_A_ receptor.

Since we noted a significant anticonvulsant effect of schisandrin B in the zebrafish larval PTZ seizure test, we used this animal model for testing of schisandrin B toxicity, i.e., its influence on zebrafish development and early life-stage swimming behavior. The zebrafish embryo acute toxicity test (Test No. 236) was employed to assess the safety of schisandrin B after 95 h-long exposure, starting at 1 hpf embryos [90], and the light–dark transition and thigmotaxis test were employed to evaluate the changes in their behavior. A zebrafish embryo acute toxicity study, endorsed by the Organization for Economic Cooperation and Development (OECD), is a guideline for the testing of chemicals [90]. The light–dark and thigmotaxis tests were not only used in toxicological studies [91], but also as valuable tools for the assessment of anxiety-like behavior [92]. An increase in locomotor activity induced by the transition from light to dark, as well as staying or moving close to the borders of the space available (“wall hugging” or thigmotaxis), are a well-validated index of anxiety in different animals, including both adult and larval zebrafish [91,92,93]. Our toxicological studies revealed that schisandrin B provoked severe developmental changes in the zebrafish larvae; however, it did not affect their behavior in the light–dark and thigmotaxis tests on mice. These results suggest that the studied compound might have teratogenic activity and might cause developmental impairments during the prenatal stage of development. However, concentrations of schisandrin B that exerted an anticonvulsant effect in the PTZ seizure zebrafish larvae assay did not cause any damage in the later stages of zebrafish development. The results obtained suggest that schisandrin B cannot be used in the prenatal period; however, it is quite safe for organisms in later periods of development.

## 4. Materials and Methods

### 4.1. Chemicals

The analytical standard of schisandrin B was purchased from Glentham Life Sciences Ltd. (Corsham, UK). Acetonitrile and dimethyl sulfoxide (DMSO) were purchased from Merck (Darmstadt, Germany; p.a.), citric acid and disodium hydrogen phosphate (Na_2_HPO_4_) from Sigma Aldrich (St. Louis, MO, USA; p.a.), and PTZ from Sanofi-Aventis (Paris, France). Distilled water was obtained from the Direct-Q3 UV apparatus (Millipore, Warsaw, Poland).

For the zebrafish experiments, schisandrin B was dissolved in DMSO to achieve a final concentration of 10 mM in stock and then diluted in zebrafish E3 medium. The final concentration of DMSO in schisandrin B solutions did not exceed 0.06%. The equivalent dose of DMSO was diluted in a E3 medium and this group served as the control group. PTZ was dissolved in E3 medium to achieve a final concentration of 60 mM in stock.

For the mouse studies, schisandrin B was suspended in 1% Tween 80 in normal saline (0.9% NaCl) and injected at a volume of 10 mL/kg. The control group received the vehicle, i.e., 1% Tween 80 in saline, at the same volume.

### 4.2. Animals and Ethical Approval

For the zebrafish embryo toxicity assay, embryos of the AB strain were obtained from the zebrafish facility at the Experimental Medicine Centre, Medical University of Lublin, Poland. For the remaining experiments, zebrafish embryos of the AB strain were raised at the zebrafish facility at the Centre for Molecular Medicine Norway (NCMM), Oslo, Norway. In both laboratories, the zebrafish embryos were kept under standard housing conditions (28.5 °C; 14 h/10 h light/dark cycle). They were maintained in E3 embryo water, i.e., medium (1.5 mM HEPES, pH 7.6, 17.4 mM NaCl, 0.21 mM KCl, 0.12 mM MgSO_4_, and 0.18 mM Ca(NO_3_)_2_).

For the experiments conducted at NCMM, larvae up to 7 dpf were used. The experiments were approved by the Norwegian Food Safety Authority experimental animal administration’s supervisory and application system (“Forsøksdyrforvatningentilsyns- og søknadssystem”, FOTS-ID 23935). For the experiments conducted in Poland, larvae up to 4 dpf were used. Ethical permission was not required for the experiments using embryos and larval zebrafish up to 120 hpf. A total of 15 µM tricaine solution was used for zebrafish euthanasia. Experiments conducted using zebrafish larvae were repeated three times and the obtained data were pooled together. 

Naïve male Swiss mice weighing 23–28 g obtained from a licensed breeder (Laboratory Animals Breeding, Warszawa, Poland) were also used in the present study. The animals were housed in polycarbonate cages under standardized conditions with ambient temperature set at 21–23 °C, relative humidity at 45–55%, a 12/12 light/dark cycle (light on at 6:00 a.m.), and free access to chow pellets and tap water. Prior to the experiments, the animals were exposed to a one-week-long acclimatization period. All experiments were performed at the same time of day (between 8:00 a.m. and 3:00 p.m.) to minimize circadian influences. Control and drug experiments were always performed on the same day to avoid day-to-day variations in convulsive susceptibility. The experimental procedures and protocols were approved by the Local Ethics Committee in Lublin, Poland (license no. 40/2020).

The experiments in all laboratories were performed in compliance with the National Institute of Health Guidelines for the Care and Use of Laboratory Animals, the European Community Council Directive of November 2010 for Care, and Use of Laboratory Animals (Directive 2010/63/EU) guidelines. All efforts were made to minimize the number of animals used, as well as their stress and suffering levels.

### 4.3. Zebrafish Toxicity Test

To assess the safety of schisandrin B on developing organisms, a zebrafish embryo acute toxicity study was conducted in accordance with the OECD guideline for the testing of chemicals (Test No. 236), as described in detail in our previous papers [90,94]. Viable and completely transparent embryos were selected one hour after fertilization and exposed to various concentrations of schisandrin B (0.375, 0.75, 1.5, 3, or 6 µM) for 1–96 hpf, with fresh solutions provided every 24 h. The control group was exposed to 0.06% DMSO. At first, we tested the toxicity of schisandrin B at log concentrations, i.e., 1000, 100, 1, and 0.1 µM. If we found 5–4 dead fishes per group after a 23 h-long incubation period (from 1 to 24 hpf), we chose lower concentrations of schisandrin B until we found a dose that did not cause mortality until 96 hpf. Only the last experiments were replicated a few times using a higher number of zebrafish larvae. At 72 and 96 hpf, the hatching rate was monitored to assess developmental delay. At 96 hpf, observations were performed to detect abnormalities, including pericardial edema, jaw development, yolk sac necrosis, swim bladder development, body axis shape/curvature, hemorrhage, and changes in pigmentation [95]. For imaging purposes, 96 hpf-old larvae were mounted in methylcellulose and photographed (Olympus SZ61, Olympus, Tokyo, Japan).

### 4.4. Evaluation of MTC of Schisandrin B for PTZ-Induced Seizure Assay

Four-day-old larval zebrafish were pretreated with schisandrin B (12, 6, 3, 1.5, or 0.75 µM) solutions for 24 h. After this time, the larvae were individually checked under the microscope (Olympus SZ61, Tokyo, Japan) to detect signs of toxicity or abnormalities. The morphological traits were scored as previously described [25,26]. In addition, escape response upon a touch of the tail (to determine muscle function) and hypoactivity (to determine sedation) were checked.

### 4.5. PTZ-Induced Seizure Assay in Larval Zebrafish

The locomotor activity experiments were conducted as described in detail in our previous papers [25,32,96]. Zebrafish larvae at 6 dpf were pretreated with schisandrin B (concentrations of 0.75, 1.5, or 3 µM) for 24 h. The tested doses were determined taking into account the MTC and results obtained in the subsequent trials. The aim was to determine the lowest doses of schisandrin B that had an anticonvulsant effect. Prior to a vehicle (control group) or PTZ application, the larvae were habituated to the behavior tracker for 15 min. Then, the vehicle or PTZ (final concentration of 20 mM) was added to each well of the 48-well plate. Five min later, larval activity was monitored (ZebraBox, Viewpoint, Lyon, France) for 30 min with 2 min time intervals. The distance covered by each larva in millimeters (mm) was measured. The data from three independent experiments were pooled together.

### 4.6. LFP Recordings in Larval Zebrafish

The LFP recordings were taken as described in our previous papers [25,32,97]. Six-day-old larvae were pretreated with schisandrin B (3 µM, the highest tested dose in the zebrafish larvae PTZ assay) or vehicle for 24 h with 5 min-long follow-up exposure to 20 mM PTZ. Then, the larvae were mounted on a glass slide in a layer of low-melting-point agarose (2%). Subsequently, the glass electrode filled with artificial cerebrospinal fluid (124 mM NaCl, 2 mM KCl, 2 mM MgSO_4_, 2 mM CaCl_2_, 1.25 mM KH_2_PO_4_, 26 mM NaHCO_3_, 10 mM glucose) was inserted into the optic tectum (MultiClamp 700B amplifier, Digidata 1550 digitizer, Axon Instruments, Scottsdale, AZ, USA). The LFP recordings for each larva were collected within a 20 min time frame. For the analysis, Clampfit 10.2 software (Molecular Devices Corporation, San Jose, CA, USA) and custom-written R script for Windows were used.

### 4.7. RT-qPCR Assessment

RT-qPCR analysis was conducted according to our previously published methods [32]. Six-day-old larvae were pretreated with schisandrin B (3 µM, the highest tested dose in the zebrafish larvae PTZ assay) or vehicle for 24 h with 90 min-long follow-up exposure to 20 mM PTZ. After the treatment, the larvae were collected in a pool of *n* = 10/sample and RNA was extracted using TRIZOL (Invitrogen, Carlsbad, CA, USA). Subsequently, cDNA was synthesized using SuperScript™ IV First-Strand Synthesis System (Invitrogen, Carlsbad, CA, USA) and amplified using PowerUp™ SYBR™ Green Master Mix (Applied Biosystems, Waltham, MA, USA), following the manufacturer’s instructions. Relative enrichment was computed according to the 2^−ΔΔt^ method, while expression levels were normalized against the housekeeping gene glyceraldehyde 3-phosphate dehydrogenase (*gapdh*) [32]. Primer sequences:

(1) gapdh_f_5′GTGGAGTCTACTGGTGTCTTC3′;

(2) gapdh_r_5′GTGCAGGAGGCATTGCTTACA3′;

(3) c-fos_f_5′CCGATACACTGCAAGCTGA 3′;

(4) c-fos_r_ 5′TGCGGCGAGGATGAACTCTA3′;

(5) bdnf_f_5′AGCTGAAGAGACAACTTGC3′;

(6) bdnf_r_5′CCATAGTAACGAACAGGAT3′.

### 4.8. Light–Dark Transition and Thigmotaxis Tests

The light–dark transition and thigmotaxis tests were performed, as previously described [97]. Briefly, 7 dpf larvae pretreated in schisandrin B (at the same doses as in the zebrafish larvae PTZ test, i.e., 0.75, 1.5, and 3 µM) for 24 h were placed individually in a 24-well plate. The larvae were habituated in the dark for 15 min. Then, they were tracked 10 min each in alternating light, dark, light and dark illumination conditions. For the XY graph, locomotor activity for 1 min time bins was extracted. Locomotor activity in each illumination condition was reported as the total distance, which was the sum of small and large movements. The thigmotactic behavior of the larvae in each illumination condition was calculated as a percentage of the total distance traveled in the outer zone of the arena. The data were pooled from three independent experiments performed on the same day.

### 4.9. Evaluation of Schisandrin B Concentration in the Plasma and Brain of Mice

The concentration of schisandrin B was measured in plasma samples and brain homogenates using an HPLC-ESI-QTOF-MS/MS platform (Agilent Technologies, Santa Clara, CA, USA) in the tailored quantitative experiment. First, the serum samples (three samples per group) were mixed with acetonitrile in the ratio of 1:1 *v/v*, vortexed for 10 min, centrifuged at 12,000 rpm for 30 min, and filtered through a nylon syringe filter with a pore-size diameter of 0.1 µm. The collected brains (three organs per group) were homogenized with 300 µL of acetonitrile:water (50:50 *v/v*) using a tissue homogenizer (PRO 200, PRO ScientificInc., Oxford, CT, USA). They were centrifuged at 12,000 rpm for 30 min and the supernatant was filtered through nylon syringe filters with a pore diameter of 0.1 µm to the autosampler vials. Directly after the preparation of the samples, they were introduced to the HPLC-MS instrument. For the analysis, the HPLC chromatograph (1200 Series) equipped with a degasser (G1322A), binary pump (G1312C), autosampler (G1329B), and mass detector (G6530B) with electrospray ionization was used (Agilent Technologies, Santa Clara, CA, USA). The chromatographic separation of the matrix was achieved on a Zorbax Eclipse Plus RP-18 (150 mm × 2.1 mm; dp = 3.5 um) column, in a gradient of acetonitrile with 0.1% of formic acid (solvent A) in a 0.1% aqueous solution of the acid (solvent B) in the following program: 0 min: 98% B; 5 min: 80% B; 40 min: 75% B; 45–49 min: 30% B, 50 min: 2%. All solvents were of LC-MS grade. The analysis time was set at 60 min, the flow rate at 0.2 µL/min, the temperature at 20 °C, and the injection volume at 10 µL. The analysis was recorded in both positive and negative ionization modes, in the following settings of the mass spectrometer: capillary voltage—3000 V, gas and sheath gas temperatures—275 and 300 °C, collision energies—10 and 20 V, skimmer voltage—65 V, and nebulizer pressure—35 psig. The spectra were registered in the m/z range of 50–1200 u, and the MS/MS spectra were recorded for every two major signals per scan. Agilent Mass Hunter Workstation (version B.10.00) was used to record and handle the data. The standard of schisandrin B was purchased from Glentham Life Sciences Ltd. (Corsham, UK) and was used for the preparation of a calibration curve equation and the following quantitative analysis of schisandrin B in the biological samples. The samples were injected 6 times (*n* = 6).

### 4.10. The Timed PTZ Infusion Test in Mice

The test was conducted according to the method described previously by Nieoczym et al. [96]. At the appropriate time after schisandrin B (50 mg/kg) or vehicle (1% Tween 80) administration, the mice were placed in the restrainer. To perform the infusion into the lateral tail vein, a needle (27G, ¾ in., Sterican^®^, B. Braun Melsungen AG, Melsungen, Germany) attached by a polyethylene tube (PE20RW, Plastic One Inc. Roanoke, VA, USA) with a plastic syringe containing a 1% solution of PTZ in saline and placed in the syringe pump (model Physio 22, Hugo Sachs Elektronik-Harvard Apparatus GmbH, March-Hugstetten, Germany) was used. The proper placement of the needle in the vein was confirmed by the presence of blood in the tube and, then, adhesive tape was used to secure the needle to the tail. The animal was taken out of the restrainer and the PTZ solution was infused into the vein of a freely moving mouse at a constant rate of 0.2 mL/min. The time intervals from the start of the infusion of the PTZ solution to the appearance of three separate endpoints, i.e., first myoclonic twitch, generalized clonus with loss of righting reflex, and forelimb tonus, were registered. The seizure threshold (in mg of PTZ per kg of body weight) for each endpoint was calculated according to the following formula: PTZ (mg/kg) = (infusion duration (s) × infusion rate (mL/s) × PTZ concentration (mg/mL))/weight (kg).

Experimental groups consisted of 11–12 animals.

The IV PTZ test dose of schisandrin B was the same as in the experiments evaluating the changes in its concentration in the mouse brain tissue and plasma. This was determined based on our preliminary studies.

### 4.11. In Silico Studies

#### 4.11.1. Chromatographic Biomimetic Studies

The Shimadzu Vp liquid chromatographic system (Shimadzu, Kyoto, Japan) equipped with LC 10AT pump, SPD 10A UV-Vis detector, SCL 10A system controller, CTO-10 AS chromatographic oven, and Rheodyne injector valve with a 20 µL loop was applied in the HPLC measurements.

The solution of schisandrin B was prepared in DMSO at a concentration of 1 mg/mL. The chromatographic conditions were first optimized. The IAM.PC.DD2 stationary phase (100 × 4.6 mm i.d., 10 µm; Regis Chemicals Company, Morton Grove, IL, USA) was employed. Acetonitrile–phosphate-buffered solutions (0.4; 0.5; 0.6; 0.7 *v/v*; pH 7.4) were used as mobile phases.

The buffer was prepared from the solutions of Na2HPO4 (0.02 mol/dm^3^) and citric acid (0.01 mol/dm^3^), and the pH 7.4 value was fixed before the preparation of the mobile phases. The mobile phase flow rate was set at 1 mL/min. The analyte was detected with a UV light at 225 nm. All measurements were obtained at 25 °C. The dead time values were measured from the non-retained compound (citric acid) peaks. The retention time was the average of at least three independent measurements. The values of the peak asymmetry factor were in the acceptable range.

ACD/Percepta software (version 2012, Advanced Chemistry Development, Inc., Toronto, ON, Canada) was used in the in silico studies. The statistical analysis of the obtained results was performed using the Minitab 18 Statistical Software (Minitab Inc., State College, PA, USA).

#### 4.11.2. Molecular Modeling

The 3D structure of schisandrin B was modeled using a LigPrep module [98] of Schrödinger suite of software, v. 2019-4, as previously reported [99,100]. In order to sample the ligand protonation state Epik module [101] of Schrödinger suite of software, v. 2019-4 was applied.

PASS 2022 and Pharma Expert software were used to obtain possible molecular targets of schisandrin B [102]. Receptor structures were downloaded from Protein Data Bank. In the case of the dopamine D_2_ receptor, the cryo-EM structure of the human receptor in active conformation in the complex with an agonist bromoergocriptine (PDB ID: 6VMS) [103] was used. Similarly, for the serotonin 5-HT_2A_ receptor, the cryo-EM structure of the receptor in active conformation in a complex with an agonist 25-CN-NBOH (PDB ID: 6WHA) [104] was selected for molecular docking. In the case of the NMDA receptor, the cryo-EM structure of the human GluN1/GluN2A NMDA receptor in the glycine/CPP bound state (PDB ID: 7EOQ) [105] was used to further studies. Finally, for the GABA_A_ receptor, the cryo-EM structure of the human full-length heteromeric α1β3γ2L GABA_A_ in a complex with alprazolam (Xanax) and GABA (PDB ID: 6HUO) [106] was applied. The structures of the receptors were preprocessed using the Protein Preparation Wizard of Maestro Release 2019.4 [107], as previously reported [99,100]. Protonation states of the respective receptor residues were assigned. Hydrogen atoms were added, and minimization was performed, restricted to hydrogen atoms only. Yasara Structure v. 20.12.24 [108] tool for loop modeling was used to construct receptor loops, if necessary. Other missing parts of the receptors were built using Prime [109] of Schrödinger suite of software, v. 2019-4.

The Standard precision (SP) method of molecular docking with Glide [110] from Schrödinger release 2019-4 was applied. For GPCRs and GABA_A_ receptors, the grid files were obtained based on co-crystallized ligands (alprazolam in the case of GABA_A_ receptor). For the NMDA receptor, grid file was based on the phencyclidine position in the ion channel taken from the rat structure of NMDA receptor in complex with phencyclidine (PDB ID: 7SAB) [111]. The selected receptor hydroxyl groups in the active sites were made flexible for GPCRs, as previously reported [112,113]. In the case of the NMDA receptor, the hydroxyl groups of the following residues were made flexible, Thr646A, Thr648B, Thr646C, and Thr648D, while for the GABA_A_ receptor: Tyr58C, Tyr160D, Ser205D, and Ser206D. A total of 100 poses were generated in the case of each ligand–receptor complex. Final poses were selected from the highest-scoring poses based on the visual inspection. Maestro Release 2019.4 [107] and PyMol 2.0.4 [114] software were used for the visualization of the molecular modeling results. Prime MM/GBSA [115] of Schrödinger suite of software v. 2019-4 was used to estimate the relative binding affinity of the protein–ligand complex, without taking into account any simulation process. The flexible residue distance was defined for 5 Å.

### 4.12. Statistical Analysis

One-way or two-way ANOVAs with repeated measures, with Tukey’s or Bonferroni’s post hoc tests, respectively, were used to analyze locomotor activity, thigmotaxis data, LFP recordings, and RT-qPCR from the zebrafish larvae, as well as the results obtained from the IV PTZ test in mice. The results are presented as mean ± SEM, ± SD or individual measurements with mean ± SEM. In the toxicological assay, the chi-squared and Fisher’s exact tests were used for analyses. The criterion for statistical significance was set at *p* < 0.05. For statistical purposes and figure generation, GraphPad Prism 8.4.3 or 9.3.1 versions (San Diego, CA, USA) were used.

## 5. Conclusions

Our study revealed the anticonvulsant properties of schisandrin B. However, the anticonvulsant effect was observed only in the larval zebrafish PTZ seizure assay. Although schisandrin B could cross the BBB, which was verified both in in silico and in vivo studies in mice, it did not influence convulsant activity in the timed PTZ infusion test in mice. The lack of anticonvulsant action in the mouse seizure test might have resulted from a very low level of unbound fraction of the compound in brain tissue, which significantly prevented its interaction with the respective molecular targets, i.e., with GABA_A_ and/or NMDA receptors. The toxicological studies reveal that schisandrin B has a detrimental impact on zebrafish embryonic development, which suggests its teratogenic potential. Further detailed studies to clarify the properties, safety, and mechanisms of action of schisandrin B are required.

## Figures and Tables

**Table 1:** The BBB pharmacokinetic descriptors of schisandrin B calculated in silico (ACD/Percepta).

Log BB	Log PS	logPS*_fu,brain_	Fu	Fb
1.15	−1.1	−3.2	0.11	0.01

**Table 2:** Physicochemical parameters calculated in silico.

LogPow	LogPcw	MW [g/mol]	TPSA [Å^2^]	Polarizability	E
4.791	3.753	400.46	55.38	43.58	2.23

**Table 3:** Glide scores and MMGBSA results for the selected complexes of schisandrin B with the studied receptors.

No.	Receptor	Glide Score	MMGBSA dG Bind [kcal/mol]
1	D_2_	−5.341	−50.27
2	5-HT_2A_	−4.775	−33.54
3	NMDA	−7.125	−42.63
4	GABA_A_	−6.955	−49.25

## Data Availability

Not applicable.
